# Supplementary material for: Autism, ADHD, and Their Traits in Adults with Obesity: A Scoping Review
Source: Nutrients. 2025 Feb 24;17(5):787. doi: 10.3390/nu17050787 (PMC11901760; doi:10.3390/nu17050787)
Supplement: Supplementary file 1 [file nutrients-17-00787-s001.zip › nutrients-3469480-supplementary.pdf]

Autism, ADHD, and Their Traits in Adults with Obesity: A Scoping Review
Supporting information

Table S1. Eligibility criteria for papers included in this review.

**Inclusion criteria**	**Exclusion criteria**
Title and Abstract screening	
• Involved human adults (18+ years) with polygenic, non-syndromic Obesity (BMI≥30). For qualitative studies, samples of carers, family members, health care professionals, or experts were also included.<br>• Assessed for, or reported, diagnosed or suspected autism or ADHD, or autistic or ADHD traits (referred to simply as 'autism' and 'ADHD' for the rest of the criteria) in the sample.	• Studies on animals, children, non-obese samples (BMI<30), monogenic or syndromic obesity or autism were excluded. This was to ensure that literature selected is relevant to clinicians working with adult bariatric populations. Monogenic and syndromic autism and obesity are likely to have a unique relationship with each other compared to more common forms of autism and obesity.
Full-text screening	
• Reported relative prevalence of autism or ADHD in patients with Obesity; experiences or perspectives of this group and other stakeholders; clinical differences of this patient group compared to patients without autism or ADHD; or intervention options for this group.<br>• Prevalence papers had to report effect sizes (and confidence intervals) or p-values to be included.<br>• Only studies with primary data or those that analyse secondary data (such as in cohort studies).<br>• Peer-reviewed articles, pre-prints, and abstracts were all included.<br>• The sample could be of any sex or ethnicity and samples with other comorbidities were not excluded. Studies could be from any location or year.	• Prevalence papers were excluded if they only reported raw percentages, without a comparison group and without reporting effect sizes or significance testing.<br>• Studies were excluded if the target sample was aggregated with a wider sample, such as in a mixed ED sample or with children (17 and under).<br>• Studies were also excluded if there was insufficient information in the abstract for extraction, and full text was not available in English, or an English version could not easily be generated.<br>• No reviews, comment pieces, or theoretical papers. Reviews were set aside for hand-searching.<br>• Studies which only investigated unidimensional autism or ADHD traits (e.g. impulsivity) were excluded.<br>• Studies only looking at biological data were excluded, as these are unlikely to immediately inform clinical practice.

ADHD = attention-deficit/ hyperactivity disorder; BMI = Body Mass Index.

Table S2. Full search strategy for each database.

Database	Search strategy
Embase Classic + Embase	bulimia/ OR binge eating disorder/ OR obesity/ OR diabetic obesity/ OR morbid obesity/ OR sarcopenic obesity/ OR "bulimi*".kf,tw. OR “binge eating disorder*”.kf,tw. OR "obes*".kf,tw.<br>AND<br>autism/ OR asperger syndrome/ OR attention deficit hyperactivity disorder/ OR neurodiversity/ OR "autis*".kf,tw. OR "asperger*".kf,tw. OR attention deficit.kf,tw. OR adhd.kf,tw. OR “neurodiver*”.kf,tw.<br>NOT<br>((exp animal/ or exp invertebrate/ or nonhuman/ or animal experiment/ or animal tissue/ or animal model/ or exp plant/ or exp fungus/) not (exp human/ or human tissue/))
Ovid MEDLINE® ALL	bulimia nervosa/ OR binge-eating disorder/ OR obesity/ or obesity, morbid/ OR "bulimi*".kf,tw. OR “binge eating disorder*”.kf,tw. OR "obes*".kf,tw.<br>AND<br>exp Autism Spectrum Disorder/ OR Attention Deficit Disorder with Hyperactivity/ OR "autis*".kf,tw. OR "asperger*".kf,tw. OR attention deficit.kf,tw. OR ADHD.kf,tw. OR “neurodiver*”.kf,tw.<br>NOT<br>(exp animals/ not humans.sh.)
APA PsycINFO	bulimia/ OR binge eating disorder/ OR obesity/ OR "bulimi*".tw. OR “binge eating disorder*”.tw. OR "obes*".tw.<br>AND<br>exp autism spectrum disorders/ OR exp attention deficit disorder/ OR neurodiversity/ OR "autis*".tw. OR "asperger*".tw. OR attention deficit.tw. OR adhd.tw. OR “neurodiver*”.tw.
Clarivate Web of Science Core Collection	TS=(bulimi*) OR TS=(binge eating disorder*) OR TS=(obes*)<br>AND<br>TS=(autis*) OR TS=(asperger*) OR TS=(attention deficit) OR TS=(adhd) OR TS=(neurodiver*)
CENTRAL	MeSH descriptor: [Bulimia Nervosa] explode all trees OR MeSH descriptor: [Binge-Eating Disorder] explode all trees OR MeSH descriptor: [Obesity] explode all trees OR (bulimi*):ti,ab,kw OR (binge eating disorder*):ti,ab,kw OR (obes*):ti,ab,kw<br>AND<br>MeSH descriptor: [Autism Spectrum Disorder] explode all trees OR MeSH descriptor: [Attention Deficit Disorder with Hyperactivity] explode all trees OR (autis*):ti,ab,kw OR (asperger*):ti,ab,kw OR (attention deficit):ti,ab,kw OR (adhd):ti,ab,kw OR (neurodiver*):ti,ab,kw

Scopus (Elsevier)	(TITLE-ABS-KEY (bulimi*)) OR (TITLE-ABS-KEY ("binge eating disorder*")) OR (TITLE-ABS-KEY (obes*))<br>AND<br>(TITLE-ABS-KEY (autis*)) OR (TITLE-ABS-KEY (asperger*)) OR (TITLE-ABS-KEY ("attention deficit*")) OR (TITLE-ABS-KEY (adhd)) OR (TITLE-ABS-KEY (neurodiver*))

Figure S1. Number of included publications from 2001 to 2024.

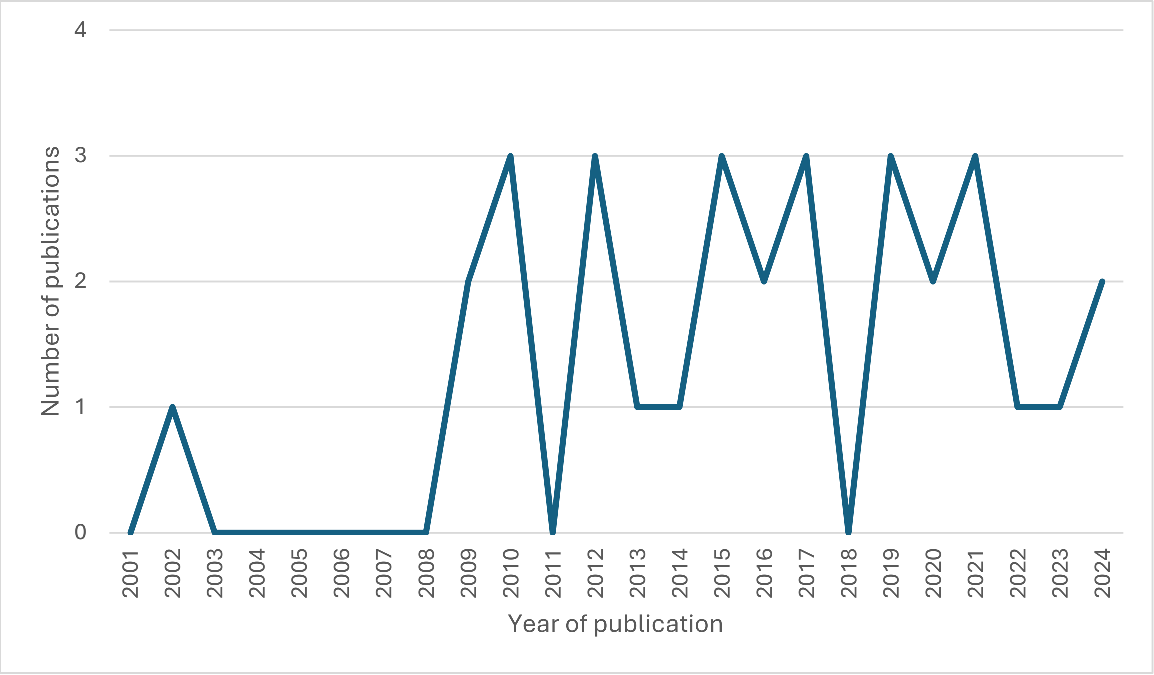

Table S3. Descriptions of studies reporting prevalence of ADHD in adults with Obesity.

**Study**	**Country, Design**	**ADHD** *(measure)*	**Sample,** *n*	**BMI** *Mean ± SD (range)*	**Age, years** *Mean ± SD (range)*	**Gender** *Women, %*	**Ethnicity** *White, %*	**Findings** *Mean ± SD/ n (%; CI)*	**Statistical tests**
Akcan et al. (2021)	Turkey, Case-control	Traits *(AADHDS)*	100 Ob patients	(30+)	(18-65)	76	-	31.69±15.51	*T*=1.60, *p*=.11
			100 non-Ob	(<25)	(18-65)	58	-	28.02±16.93	
Alfonsson et al. (2014)	Sweden, Longitudinal	Traits (*ASRS-18)*	129 Bariatric	*Pre-surgery:* 42.95±3.98	42.8±10.52	78	-	*Pre-surgery*	*Attention*
								Attention: 9.79±4.88	*F*=0.79, *p*=.38, *d*=0.16
								Hyperactivity: 9.72±4.51	
								Post-surgery	*Hyperactivity*
								Attention: 9.64±6.5	*F*=14.36, *p*<.001, *d*=0.67
								Hyperactivity: 8.18±5.92	
Altfas (2002)	USA, Retrospective	Diagnosis *(DSM-IV, clinical interview)*	215 Ob/ Ov patients	(25+)	44.6±11.4	84.7	-	*n*=59 (27.4%; 21.1-32.9%)	*Within ADHD diagnosis*
			61 Ob-III	(40+)	-	-	-	*n*=26 (42.6%; 36.3-48.9%)	*Ob-III vs Ob-I/II/ Ov* χ^2=9.86, *df*=1, *p*=.002
			101 Ob-I/II	(30-39)	-	-	-	*n*=23 (22.8%; 14.6-31.0%)	*Ob-I/II/III vs Ov* $\chi^2$2.60, *df*=1, *p*=.11
			53 Ov	(25-29)	-	-	-	*n*=10 (18.9%; 8.4-29.4%)	
		Traits *(3+ symptoms)*	215 Ob/ Ov patients	(25+)	42.7±91.7	91.7	-	*n*=72 (33.5%; 27.2-39.8%)	*ADHD diagnosis, traits, vs no ADHD*
			61 Ob-III	(40+)	-	-	-	*n*=18	*Across Ob-III, Ob-I/II & Ov*
			101 Ob-I/II	(30-39)	-	-	-	*n*=38	
			53 Ov	(25-29)	-	-	-	*n*=16	χ^2=11.23, *df*=4, *p*<.025
		No diagnosis or traits	215 Ob/ Ov patients	(25+)	43.4±10.9	91.7	-	*n*=84 (39.1%; 32.6-45.6%)	
Davis et al. (2009)	Canada, Case-double control	Traits *(CAARS-SSV, WURS-25)*	60 Ob patients	39.2±8	37.0±6.7	75.0	72.4	*CAARS:* 13.4±5.7	*Ob vs BE vs NW*
								Attention: 8.6±5	*CAARS:* χ^2=26.27, *df=2*, *p*<.0001
								Hyperactivity: 8.3±4.9	*Ob vs BE: Wald=1*, *df*=1, *p*=.317
								WURS: 34.3±19.9	
			61 BE patients	34.7±8.6	34.5±6.5	78.3	81.4	*CAARS:* 14.5±6	*BE vs NW: Wald=19.74, df*=1, *p*<.0001
								Attention: 9.6±4.7	
								Hyperactivity: 9±4.2	
								WURS: 34.8±20.6	*WURS:* χ^2=27.66, *df=2*, *p*<.0001
			60 NW	22.4±2.7	33.3±7.5	88.5	82.0	*CAARS:* 9.3±5.1	
								Attention: 6.8±3.8	*Ob vs BE : Wald=.01, df*=1, *p*=.907
								Hyperactivity: 6.8±3.7	

								WURS: 18.1±16.3	*BE vs NW :* *Wald=18.12, df*=1, *p*<.0001
Docet et al. (2010)	Spain, Case-control	Diagnosis *(DSM-IV-TR, ASRS-6)*	170 Ob patients	(30+)	41±13.4 (18-79)	75.9	-	*n*=35 (20.6%), 7 previously diagnosed	*Ob vs normal*
									χ^2=7.010, *df*=1, *p*=.008, OR=3.5
								Female	*Within Ob*
								(24%)	*Female vs Male*
								Male	χ^2=3.878, *df*=1, *p*=.049
								(9.7%)	
			67 Ob-III	(40+)				(16.4%)	*Across Ob classes*
			55 Ob-II	(35-39)				(27.3%)	χ^2=2.315, *df*=2, *p*=.314
			48 Ob-I	(30-34)				(18.7%)	
			73 non-Ob	(19-24)	41.4±12.8 (18-72)	71.2	-	*n*=5 (6.8%), 0 previously diagnosed	
								Female	*Within non-Ob*
								(7.7%)	*Female vs Male*
								Male	χ^2=0.201, *df*=1, *p*=.654
								(4.8%)	
Nazar et al. (2016)	Brazil, Cross-sectional	Diagnosis *(DSM-IV, K-SADS)*	106 Ob patients	39.21±5.29 (30+)	38.99±10.74 (18-59)	100	-	*n*=30 (28.3%; 23.78-32.82%)	*Across Ob classes*
									χ^2=0.591 *p*>.05
			Ob-III	(40+)		100	-	(13.2%)	
			Ob-II	(34-39)		100	-	(9.4%)	
			Ob-I	(30-34)		100	-	(5.7%)	
Nielsen et al. (2017)	Germany, Comparative cross-sectional	Screening, traits *(CAARS-SSV, WURS-k)*	120 Bariatric (candidates)	47.76±7.41	40.97±11.46	79.2	-	*CAARS*	*Across pre- and post-surgery groups*
								n=23 (19.2%); 54.92±13.03	*Screening*
								WURS-k	*CAARS:* χ^2=0.542, *df*=1, *p*=.462, ϕ=-0.047
								n=21 (17.5%); 17.07±14.61	
								CAARS + WURS-k	*WURS:* χ^2=0.158, *df*=1, *p*=.691, ϕ=-0.025
								n=10 (8.3%)	
			128 Bariatric (post-surgery)	*Pre-surgery:* 48.82±6.43	41.53±10.67	78.9	-	*CAARS*	
								n=20 (15.6%); 51.84±12.83	*CAARS + WURS-k:* χ^2=0.514, *df*=1, *p*=.773, ϕ=0.046
								WURS-k	*Traits*
								n=20 (15.6%); 15.94±13.05	*CAARS: t*=1.876, *df*=246, *p*=.062, *d*=-0.238
								CAARS + WURS-k	

								n=8 (6.3%)	*WURS: t*=0.639, *df*=238.55, *p*=.523, *d*=-0.082
Stahel et al. (2019)	Canada, cohort	Diagnosis *(clinical interview, MINI, prior diagnosis)*	149 Ob-IV patients	62.3±0.7 (50+)	46.4±0.9	76.5	79.2	*n*=4 (2.7%)	OR 1.46 (CI: 0.47-4.58), *p*=.18
			131 Ob-II/III patients	43.3±0.3 (35-50)	45.9±0.9	79.4	74.8	*n*=1 (0.8%)	

AADHDS= Adult ADHD Scale
ADHD= attention-deficit/ hyperactivity disorder
ASRS-6=6-item Adult ADHD Self-report Scale
BMI= Body Mass Index, kg/m^2
CAARS-SSV= Conners' Adult ADHD Rating Scale – Self-Report, Short Version
CI= 95% confidence interval
d= Cohen's d
df= degree of freedom
DSM-IV= The fourth edition of the Diagnostic and Statistical Manual of Mental Disorders
DSM-IV-TR= The text revision of DSM-IV
K-SADS= Schedule for Affective Disorders and Schizophrenia
MINI= Mini-International Neuropsychiatric Interview
n= number of participants
Ob-I/II/III/IV= Obesity classes
OR= odds ratio
Ov= Overweight
SD = standard deviation
USA= United States of America
vs = versus.
WURS-k= 25-item Wender Utah Rating Scale.
χ^2= chi-squared

Table S4: Descriptions of studies reporting differences in clinical profiles of ADHD adults compared to non-ADHD adults with Obesity.

Study	Country, Design	ADHD *(measure)*	Ob Sample	ADHD present, *n*	BMI *Mean ± SD (range)*	Age, years	Gender *Women, %*	Ethnicity *White, %*	Pre-intervention comparisons: Measures/ outcomes	Findings
Alfonsson et al. (2012)	Sweden, Cross-sectional	Screening *(ASRS-18)*	217 Bariatric candidates	Yes, 19<br>No, 168	44.28±6.02	41.04±11.07	73.3	-	HADS anxiety, depression; GFCQ-T total, loss of control, emotional cravings	ADHD>non-ADHD
Alfonsson et al. (2013)	Sweden, Cross-sectional	Screening *(ASRS-18)*	276 Bariatric candidates	Yes, 21<br>No, 224	43.96±5.82 (33.6-66.0)	42.38±11.04	72.7	-	BMI	N.S
									AUDIT, GFCQ-T, HADS	ADHD>non-ADHD
Brancati et al. (2022)[†]	Italy, Cross-sectional	Screening *(ADHD-SCL-90-R)*	110 Bariatric candidates	Yes, 14<br>No, 96	(30+)	"Adults"	-	-	BITE, NEQ	ADHD>non-ADHD
Brancati et al. (2024)	Italy, Cross-sectional	Screening *(ADHD-SCL-90-R)*	*All*							
			260 Bariatric candidates	Yes, 30	47.49±8.02	45.53±9.88	76.7	-	Age, gender, BMI, any mood disorders, family history of mood or anxiety disorders, any eating disorders (SCID-I; DSM-IV-TR)	N.S
				No, 230	46.11±7.38	44.15±10.83	71.3	-		
									Any anxiety disorder, panic disorder (SCID-I; DSM-IV-TR); psychopathological symptoms (SCL-90-R)	ADHD>non-ADHD
			Sub-group							
			95 Bariatric candidates	Yes, 13<br>No, 82	46.69±6.8	43.43±10.47	75.8	-	NEQ total, morning anorexia, evening hyperphagia, nocturnal ingestions; BITE severity scale; TCI novelty seeking, reward dependence, persistence, self-transcendence	N.S
									NEQ mood/ sleep; BITE symptom scale; TCI harm avoidance	ADHD>non-ADHD
									TCI self-directedness, cooperativeness	ADHD<non-ADHD
Brunault et al. (2019)	France, Cross-sectional	Diagnosis *(DIVA 2.0)*	105 Patients (Ob-II)	*Adult ADHD*						
				Yes, 28	46.3±88.2	46.6±11.7	85.7	-	BMI current, maximal; previous bariatric surgery, age of onset, current age, gender, sleep apnea	N.S
				No, 77	47.1±7.7	46.7±10.3	87	-		
									childhood ADHD, BES, YFAS 2.0	ADHD>non-ADHD
				Childhood ADHD						
				Yes, 37	46.6±8.2	46.4±11.6	83.8	-	BMI current, previous maximal; previous bariatric surgery, age of onset, current age, gender, sleep apnea	N.S
				No, 68	47.1±7.6	46.6±10.2	88.2	-		

									adult ADHD, BES, YFAS 2.0	ADHD>non-ADHD
Dempsey et al. (2010)	USA, Cross-sectional	Screening *(ASRS-6)*	125 Community volunteers	Yes, 27	40.27±5.28	43.67±12.19	65.6	85.6	Age, education, BMI, ST	N.S
				No, 98	38.75±6.57	43.69±13.87			BIS-11; EES; EI Disinhibition, Hunger	ADHD>non-ADHD
									EI Cognitive restraint	ADHD<non-ADHD
Dickinson et al. (2024)	USA, Retrospective case-control cohort	Diagnosis *(ICD-10: F9.0)*	*Unmatched*							
			996,929 Patients	Yes, 53,740	34.2±4.7	44.0±12.3	60.7	80.6	Ethnicity (% white); Received pharmacotherapy, surgery, orlistat, phentermine-topiramate, naltrexone-bupropion, GLP-1 agonists, short-term stimulants;	ADHD>non-ADHD
				No, 943,189	34.7±5.0	53.3±12.6	62.5	64.2	Age, gender (% female), BMI	ADHD<non-ADHD
			Matched							
			107,422 Patients	Yes, 53,711	34.2±4.7	44.0±12.3	60.8	80.6	Received pharmacotherapy, surgery, orlistat, phentermine-topiramate, naltrexone-bupropion, GLP-1 agonists, short-term stimulants;	ADHD>non-ADHD
				No, 53,711	34.2±4.7	44.0±12.3	61.2	80.9		
			5-year cohort							
			56,754 Patients	Yes, 4,129	*Pharmacology group:* 36.2±6.4	-	-	-	BMI pre-surgery	ADHD>non-ADHD
					Surgery group: 42.4±5.6	-	-	-	BMI pre-pharmacotherapy	ADHD<non-ADHD
				No, 52,625	*Pharmacology group:* 37.2±6.1	-	-	-		
					Surgery group: 41.9±6.0	-	-	-		
Docet et al. (2012)	Spain, Case-control	Screening *(ASRS-18)*	230 Patients	Yes, 51	40.3±5.7	42.3±15.5 (18-76)	88.2	-	EPQ Eating large amounts of food	N.S
				No, 179	40.6±5.9	50.9±12.4 (19-79)	78.2	-	EPQ Between meal snacking, binge eating, nighttime eating, secret eating	ADHD>non-ADHD
El Archi et al. (2021)	France, Cross-sectional	Screening *(ASRS-18, WURS-25)*	282 Bariatric candidates	Yes, 23	47.0±11.8	40.7±12.3	76.6	-	Age; BMI; BFI extraversion, openness	N.S
				No, 259	45.3±7.3	43.4±11.1			BES; YFAS 2.0; WURS-25; DERS-16; TAS-20; BFI neuroticism	ADHD>non-ADHD
									BFI agreeableness, conscientiousness	ADHD<non-ADHD
Gruss et al. (2012)	Germany, Cross-sectional	Screening *(WURS-k; ADHD-SR)*	116 Bariatric candidates (Ob-III)	Yes, 14	49.8±5.8	34.8±9.7	85.7	-	Age, gender, BMI, EES, any psychiatric disorder (Axis 1 DSM-VI), BED, any depressive disorder	N.S
				No, 102	48.5±8.3	39.1±10.5	71.6	-		
									BDI, unemployed, past psychotherapy	ADHD>non-ADHD

									Educational level	ADHD<non-ADHD
Marchesi et al. (2017)	Brazil, Retrospective	Screening *(ASRS-18)*	40 Bariatric candidates	Yes, 15	47.09±6.75	48.3±10.2	93	25	Number of comorbidities	N.S
				No, 25	42.03±4.98				BMI pre-surgery	ADHD>non-ADHD
Nazar et al. (2016)	Brazil, Cross-sectional	Diagnosis *(K-SADS, DSM-IV)*	106 non-surgery patients	Yes, 30	38.45±4.24	38.26±11.33	100	-	Age, BMI, STAI-(T)	N.S
				No, 76	39.51±5.65	39.26±10.57	100	-	BDI, BES, BITE	ADHD>non-ADHD
Nicolau et al. (2015)	Spain, Case-control	Screening *(ADHD-S)*	60 Bariatric candidates	Yes, 19	45.84±4.39	46.8±8.6	78.9	-	BMI pre-surgery; surgery type	N.S
				No, 41	49.51±8.31	46.1±10.5	78	-	Psychiatric comorbidities (BDI-S+EHQ-ES)	ADHD>non-ADHD
Pagoto et al. (2010)	USA, Cross-sectional	Screening *(ASRS-18)*	63 patients	Yes, 19	40.75±6.51	49±10.3	75	99	BMI, pre-intervention, depression (DSM-IV), type-2 diabetes, Weight loss attempts lasting >3 days	N.S
				No, 44	41.67±6.94					
									Weight loss attempts lasting <3 days	ADHD>non-ADHD
Sahan et al. (2021)	Turkey, Case-control	Diagnosis *(SCID-5, ASRS-18, WURS-25)*	100 Bariatric candidates	Yes, 50	45.35±5.21	35.59±12.12	78	-	EI total, disinhibition of eating control	N.S
				No, 50	44.81±4.88	34.46±10.54	66	-		
									EI emotional eating, susceptibility to hunger; BDI; BAI	ADHD>non-ADHD
									EI cognitive restraint of eating	ADHD<non-ADHD
Schafer et al. (2020)	Germany, Cross-sectional	Screening *(ADHD-SR)*	78 patients/ general population	Yes, 17	48.1±8.3 (33.8-78.9)	42.9±10.4 (24-69)	66.7	-	CLT NAD	ADHD<non-ADHD
				No, 61						
Stenberg et al. (2023)	Sweden, Cohort case-control	Diagnosis *(ICD-10 prescriptions, N06BA)*	4,293 patients	Yes, 1,431	41.8±5.6	34.8±11.1	75.3	>88.7	*Matched pairs:* OP (HRQoL); *Unmatched pairs:* sleep apnea	ADHD>non-ADHD
				No, 2,862	41.8±5.7	35.0±5.7	76.7	-		
									Matched: SF-36 (HRQoL); *Unmatched:* education; income	ADHD<non-ADHD
Taymur et al. (2015)†	Turkey, Cross-sectional	Screening *(WURS-25)*	89 Bariatric candidates (Ob-III)	Yes, 13	46.42±5.34	34.84±9.93	77.5	-	EAT-40; SCL-90 somatization, anxiety	N.S
				No, 76						
									SCL-90 obsessive compulsive, interpersonal sensitivity, depression, hostility, phobic anxiety, paranoid ideation, psychoticism, additional scale	ADHD>non-ADHD
Taymur et al. (2016)	Turkey, Cross-sectional	Screening *(WURS-25)*	177 Bariatric candidates (Ob-III)	Yes, 34	(40+)	36.60±8.46	80.8	-	ASRS-18, SCL-90	ADHD>non-ADHD
				No, 143						

†Abstract-only, sample may overlap with study directly below

ADHD = attention-deficit/ hyperactivity disorder

ADHD-SR = German ADHD Rating Scale

ASRS-6 = short version of the Adult ADHD Self-Report Scale

ASRS-18 = full version of the Adult ADHD Self-Report Scale

AUDIT = Alcohol Use Disorders Identification Test
BAI = Beck's Anxiety Inventory
BDI = Beck's Depression Inventory
BES = Binge Eating Scale
BFI = Big Five Inventory
BIS-11 = Barratt Impulsiveness Scale Version 11
BITE = Bulimic Investigatory Test, Edinburgh
BMI = Body Mass Index, kg/m^2
CLT NAD = Card and Lottery Task, Number of Advantageous Decisions
DERS-16 = Difficulties in Emotion Regulation Scale
DIVA 2.0 = 2nd version of the Diagnostic Interview for ADHD in adults
DSM-IV(-TR) = Diagnostic and Statistical Manual of Mental Disorders, Fourth Edition (Text Revision)
EAT-40 = Eating Attitude Test
EES = Emotional Eating Scale
EI = Eating Inventory
EPQ = Eating Pattern Questionnaire
F9.0 = ICD-10 code denoting ADHD
GFCQ-T = General Food Cravings Questionnaire-Trait
HADS = Hospital Anxiety and Depression Scale
HRQoL = Health-Related Quality of Life
ICD-10 = International Classification of Diseases, 10th Revision
K-SADS-E = Schedule for Affective Disorders and Schizophrenia module for ADHD, adapted for adults
N = number of participants
N.S = no significant differences between ADHD and non-ADHD participants
N06BA = ICD-10 code denoting Central Nervous System Stimulants, primarily medications used for ADHD, such as methylphenidate and amphetamine derivatives
NEQ = Nighttime Eating Questionnaire
Ob = Obesity
Ob-II = BMI>=35
Ob-III = BMI>=40
OP = Obesity-related Problems scale
SCID-5 = Structured Clinical Interview for DSM-5 Clinician Version
SCL-90-R = the Symptom Checklist-90-Revised
SD = standard deviation
SF-36 = Short Form Health Survey
ST = Stroop Test
STAI = State-Trait Anxiety Inventory
TAS-20 = Toronto Alexithymia Scale-20
TCI = Temperament and Character Inventory
USA = United states of America
WURS-25 = short-version of the Wender Utah Rating Scale
WURS-k = German short-version of the Wender Utah Rating Scale
YFAS 2.0 = Yale Food Addiction Scale

Table S5. Descriptions of studies reporting differences in response to Obesity treatment in ADHD adults compared to non-ADHD adults.

Study	Country, Design	Sample, *n*	ADHD *(measure)*	Intervention	ADHD present, *n*		Pre-Intervention BMI, *Mean ± SD*	Age, years *Mean ± SD*	Gender *Women, %*	Ethnicity *White, %*	Post-intervention comparisons	
											**Measures/ outcomes**	**Findings**
Alfonsson et al. (2014)	Sweden, Longitudinal	129	Screening *(ASRS-18)*	Surgery (RYGB)	*Pre-surgery*		42.95±3.98	42.8±10.52	78	-	%EBMIL	N.S (pre- or post-surgery ADHD)
					Yes	9						
					No	120						
					Post-surgery						AUDIT, HADS	Post-surgery ADHD>post-surgery non-ADHD
					Yes	10						
					No	119						
Altfas (2002)	USA, Retrospective	61	Diagnosis, traits *(DSM-IV, clinical assessment)*	Unspecified, likely behavioural	*Diagnosis*		40+	-	-	-	BMI loss, weight loss	ADHD diagnosis <ADHD symptoms <non-ADHD
					Yes	26						
					Traits							
					Yes	18						
					Diagnosis, traits							
					No	17						
Dickinson et al. (2024)	USA, Retrospective case-control cohort	56,754	Diagnosis *(ICD-10: F9.0)*	Pharmacology	Yes	2,977	36.2±6.4	-	-	-	BMI loss post-surgery (years 1-5)	ADHD>non-ADHD
					No	38,708	37.2±6.1					
				Surgery (inc. RYGB, other GBPs)	Yes	1,152	42.4±5.6				BMI loss post-pharmacology (years 1-5)	ADHD<non-ADHD
					No	13,917	41.9±6					
Laggeros et al. (2020)	Sweden, Cohort	22,539	Diagnosis *(ICD-10: F9.0)*	Surgery (GBP)	Yes	314	-	41.3±11.0	75.3	-	Reoperation within 30 days	N.S
					No	13,059					Post-operative hospital stay length	ADHD>non-ADHD
Marchesi et al. (2017)	Brazil, Retrospective	40	Screening *(ASRS-18)*	Surgery (RYGB)	Yes	15	47.09±6.75	48.3±10.2	93	25	%BMIL 3, 12 months; weight loss 12 months; success 12 months	N.S
					No	25	42.03±4.98					
											BMI 3, 6, 12 months	ADHD>non-ADHD
											%BMIL 6 months	ADHD<non-ADHD
Nicolau et al. (2015)	Spain, Case-control	60	Screening *(ASRS-18)*	Surgery (mostly RYGB)	Yes	19	45.84±4.39	46.8±8.6	78.9	-	Gender; age; unemployed; marital status; education; current BMI; EDs; BDI; SF-36 mental health, bodily pain, vitality, social functioning;	N.S
					No	41	49.51±8.31	46.1±10.5	78	-		
											% lipids intake; mono/ polyunsaturated fat; alcohol intake; grazing;	ADHD>non-ADHD
											%carbs intake; mealtime length; SF-36	ADHD<non-ADHD

											general health; adherence to protocol	
Pagoto et al. (2010)	USA, Cross-sectional	63	Screening *(ASRS-18)*	16-week clinic-based behavioural weight loss program	Yes	19	40.75±6.51	49±10.3	75	99	Meals skipped per week, physical activity (WALI)	N.S
					No	44	41.67±6.94				fast food meals per week, emotional eating (WALI); WL perceived difficulty index (PDI)	ADHD>non-ADHD
											% Weight loss; % success; eating self-efficacy (WEL)	ADHD<non-ADHD
Stenberg et al. (2023)	Sweden, Cohort matched case-control	4,293	Diagnosis *(ICD-10 prescriptions, N06BA)*	Surgery (RYGB, sleeve gastrectomy). *ADHD patients were also pharmacologically medicated.*	Yes	1,431	41.8±5.6	34.8±11.1	75.3	-	Any intra-operative complications; Specific post-operative complications; Any serious post-operative complications; Weight loss	N.S.
					No	2,862	41.8±5.7	35.0±5.7	76.7	-	Any post-operative complications; OP 1, 2 years (HRQoL); self-harm; SUD;	ADHD>non-ADHD
											RAND-36 PCS, MCS 1 year (HRQoL); RAND-36 MCS 2 years (HRQoL);	ADHD<non-ADHD

%EBMIL= percentage of excess BMI lost
ADHD = attention-deficit/ hyperactivity disorder
ASRS-18 = full version of the Adult ADHD Self-Report Scale
AUDIT = Alcohol Use Disorders Identification Test
BDI = Beck's Depression Inventory
BMI = Body Mass Index, kg/m^2
DSM-IV = Diagnostic and Statistical Manual of Mental Disorders, Fourth Edition
F9.0 = ICD-10 code denoting ADHD
GBP = Gastric Bypass Surgery
HADS = Hospital Anxiety and Depression Scale
HRQoL = Health-Related Quality of Life
ICD-10 = International Classification of Diseases, 10th Revision
n = number of participants
N06BA = ICD-10 code denoting Central Nervous System Stimulants, primarily medications used for ADHD, such as methylphenidate and amphetamine derivatives
RYGB = Roux-en-Y Gastric Bypass
SD = standard deviation
USA = United States of America
WALI = Weight and Lifestyle Index
WEL = Weight Lifestyle Self-Efficacy

Table S6: Descriptions of case reports and one intervention study.

Study	Country	Sample *n*, ethnicity, gender	Age, years	Pre-Intervention BMI	Autism/ ADHD diagnosis	Comorbidities	Medications at time of intervention	Intervention	Findings
Azran et al. (2017)	Israel	1 male	52	-	ADHD	-	Oral methylphenidate	Switched to transdermal methylphenidate after oral methylphenidate ceased working post-RYGB surgery	Therapeutic effects of methylphenidate were regained
Crowley et al. (2015)	USA	2 female	19	31.7	Autism	Cyclothymic disorder,	Bupropion, methylphenidate, guanfacine, divalproex sodium, quetiapine	Topiramate 75mg	After 17.5 months, BMI=17.8 and Topiramate use was discontinued
			20	33.1	Autism	Cyclothymic disorder, mild intellectual disability	Fluvoxamine, metformin, norgestimate, ethinyl oestradiol	Topiramate 400mg	After 24 months, BMI=23.4. Metformin and fluvoxamine were discontinued
Jarvinen et al. (2019)	Finland	1 male	20	34.4	Autism	Moderate intellectual impairment, OCD symptoms,	Aripiprazole, citalopram,	Liraglutide 2.4mg	Drastically subsided obsessive food-related thoughts, craving for food, and compulsive eating. At week 25, BMI=~30
Levy et al. (2009)	Canada	72 female, 6 male	41.3 *(mean)*	42.7 *(mean)*	ADHD *(clinical interview, WURS-25, ASRS-18)*	Sleep apnea 56.4%, binge eating disorder 65.4%, mood disorder 88.4%. Satisfactory resolution of symptoms of comorbid conditions was achieved prior to intervention	-	Sixty-five participants received pharmacotherapy, almost exclusively psychostimulants (including methylphenidate). In several cases, a nonstimulant, atomoxetine, was used due to residual anxiety symptoms. Thirteen patients remained as controls as they elected not to receive medication, had side effects, or obtained no clear benefit from trials of several medications for ADHD.	After an average of 466±260 days, weight change in treated subjects was -12.36% of initial weight and in controls +2.78%, p<0.001. Weight loss in treated subjects was 15.05kg (10.35%) and weight gain 3.26 kg (7.03%) in controls, p<0.001.

ADHD= attention-deficit/ hyperactivity disorder
ASRS-18 = full version of the Adult ADHD Self-Report Scale
BMI= Body Mass Index, kg/m^2
n= number of participants
OCD= obsessive compulsive disorder
RYGB = Roux-en-Y Gastric Bypass
USA = United States of America
WURS-25 = short-version of the Wender Utah Rating Scale
